# Sickle cell anemia and early stroke detection and prevention in Nigeria

**DOI:** 10.3389/fstro.2024.1368576

**Published:** 2024-06-19

**Authors:** Kudirat Abdulkareem Ahmed, Halima Bello-Manga, Lori C. Jordan

**Affiliations:** ^1^Department of Pediatrics, Barau Dikko Teaching Hospital, Kaduna State University, Kaduna, Nigeria; ^2^Department of Hematology and Blood Transfusion, Barau Dikko Teaching Hospital, Kaduna State University, Kaduna, Nigeria; ^3^Department of Pediatrics, Division of Pediatric Neurology, Vanderbilt University Medical Center, Nashville, TN, United States

**Keywords:** sickle cell anemia, stroke, stroke prevention, transcranial doppler, hydroxyurea, low- middle income setting

## Abstract

Sickle cell disease (SCD) is the most common hereditary blood disorder worldwide, and sickle cell anemia (SCA), the homozygous state of SCD, is the most common and severe variant of the disease. Nigeria has the highest burden of SCA in the world. Hemolysis and vaso-occlusion can lead to a wide range of complications, including stroke which is one of the most devastating manifestations of SCA with significant morbidity and mortality. SCA remains the leading cause of stroke in black children. Without any intervention, strokes occur in approximately 11% of children with SCA before their 20th birthday, with the greatest risk in very young children between 2 and 5 years of age. In resource-constrained countries, where the burden of SCA is highest, stroke is underreported, hence the need to develop strategies for stroke prevention and early detection. Improving awareness among healthcare providers and the community can significantly reduce stroke rates and improve stroke detection. The goal of this manuscript is to discuss the progress that has been made in stroke prevention and detection in children with SCA in Nigeria and outline current challenges and future goals. We believe that our experience will be valuable not only in Nigeria which has the highest burden of SCA globally, but also in other low- and middle-income countries.

## Introduction

### Overview of neurological complications of sickle cell anemia

Sickle cell disease (SCD) is the most common hereditary blood disorder worldwide, caused by an abnormal sickled hemoglobin molecule produced by the replacement of valine by glutamic acid in position 6 of the β-globin chain (Hamideh and Alvarez, 2013). Sickled hemoglobin (HbS), which polymerizes upon deoxygenation is the key pathophysiological feature of SCD resulting in chronic hemolysis and vaso-occlusion (Akinyanju, 1989; Omotade et al., 1998; Lanzkron et al., 2013). Sickle cell anemia (SCA), the homozygous state of SCD (HbSS), is the most common and most severe variant of the disease (Kato et al., 2018). Hemolysis and vaso-occlusion can lead to a wide range of complications, including pain, anemia, organ damage, and an increased risk of infection and stroke (Kato et al., 2018). SCA affects millions of people worldwide, with the highest prevalence in sub-Saharan Africa, where up to 75% of cases occur (Flint et al., 1998). SCA is a major public health problem (Grosse et al., 2011). In Nigeria, an estimated 25–30% (Fleming et al., 1979) of the population carries the sickle cell gene, and ~1–3% of children born annually have SCD (Nwogoh et al., 2012). Thus ~150,000 Nigerian children are born with SCD each year (Odunvbun et al., 2008). Nigeria accounts for up to 50% of infants born to SCD globally (Kato et al., 2018), the country has been described as the most sickle cell endemic country in Africa. The high prevalence of SCA in Nigeria is attributed to several factors, including a high rate of consanguineous and polygamous marriages, low health literacy regarding SCA, limited genetic counseling, and reduced access to screening and diagnostic facilities (Aygun and Odame, 2012).

Central nervous system (CNS) complications are among the most devastating manifestations of SCA with significant morbidity and mortality in both children and adults affected with the disease (Ohene-Frempong et al., 1998). SCA remains the leading cause of stroke in black children (Adams et al., 1997; Noubiap et al., 2017). Without any intervention, strokes occur in ~9% of children with SCA before their 14th birthday (Ohene-Frempong et al., 1998), about 11% (Ohene-Frempong et al., 1998) before their 20th birthday, with the greatest risk in very young children between 2 and 5 years of age (Ohene-Frempong et al., 1998).

Stroke is characterized by an acute focal injury of the CNS by a vascular cause, including cerebral infarction, intracerebral hemorrhage (ICH), and subarachnoid hemorrhage (SAH) (Sacco et al., 2013). Ischemic and hemorrhagic strokes can lead to a range of neurologic symptoms and can be life-threatening. Silent cerebral infarcts (SCIs) also known as silent strokes are infarcts seen via magnetic resonance imaging (MRI) of the brain without focal neurological symptoms that occur in about 30–37.4% of children with SCA by age 14 (DeBaun et al., 2012a; Connes et al., 2013). In children with SCA, SCIs are associated with a decline in neurocognitive function and poor performance on neuropsychological assessments (Armstrong et al., 1996).

### Pathophysiology of stroke in SCA

The pathophysiology of overt stroke in children with SCA is not completely understood but there are several interacting factors that may predispose to stroke. Overt ischemic stroke may occur from stenosis and occlusion of large cerebral arteries of circle of Willis, resulting from thickening of the intima caused by the proliferation of the fibroblasts and smooth muscle cells, splitting and fragmentation of the internal membrane, and superimposed thrombus (Connes et al., 2013). Deoxygenated sickled hemoglobin leads to polymerization, erythrocyte sickling, impaired blood rheology and the aggregation of sickled erythrocytes, neutrophils, platelets, and endothelial cells (Zhang et al., 2016). A hemolyzed cell releases free hemoglobin and arginase-1 which scavenges nitric oxide and competes for L-arginine, respectively, preventing the action and formation of nitric oxide. This contribute to oxidative stress and vascular remodeling, as arginase-1 converts arginine to ornithine, Consequently, endothelial function is impaired, limiting smooth muscle relaxation, causing vascular resistance and potentially predispose individual to ischemic stroke (Kato et al., 2007). Ischemia and reperfusion injury can lead to inflammation, promoting vaso-occlusion and adhesion to the vascular wall (Connes et al., 2013; Sundd et al., 2019). Hemorrhagic stroke in children with SCA is less common compared to ischemic stroke with an incidence of 50 per 100,000 person-years (Fox et al., 2022). Hemorrhagic stroke includes subarachnoid hemorrhage, intraventricular, and intraparenchymal hemorrhages. Most hemorrhagic strokes in individuals with SCA are idiopathic; however, when the cause is known, subarachnoid hemorrhage is the most common type of hemorrhagic stroke and is often due to intracranial aneurysm or moyamoya arteriopathy.

### Risk factors for stroke in SCA

Stroke is increasingly recognized as a significant cause of morbidity or mortality in children with SCA. A better understanding of the risk factors for stroke in children is the first step needed to improve strategies for stroke prevention, intervention, and to ultimately minimize the burden of stroke. In hemorrhagic stroke, other than moyamoya arteriopathy and brain aneurysm, hypertension, recent blood transfusion (in the last 14 days), acute chest syndrome, and treatment with corticosteroids have been shown to increase the stroke risk (Strouse et al., 2006). While low steady state hemoglobin, acute severe anemia with a high steady state white cell count increase the risk for both ischemic and hemorrhagic stroke (Ohene-Frempong et al., 1998; Wierenga et al., 2001; Debaun and Kirkham, 2016). In patients with SCA other risk factors include:

***Previous stroke***: children with SCA who have experienced a stroke are at a higher risk of having a recurrent ischemic stroke; the recurrence risk is about 67% within 2–3 years of the first stroke (Powars et al., 1978). Previous infarction is also associated with hemorrhagic stroke (Njamnshi et al., 2006).

***Age:*** the peak age of ischemic stroke occurrence in SCA is between 2 and 5 years of age (Ohene-Frempong et al., 1998; Fox et al., 2022). The risk is lower in children before 2 years of age probably due to the protective effect of fetal hemoglobin on sickling (Ohene-Frempong et al., 1998). While, with hemorrhagic stroke the risk increases with increasing age (Njamnshi et al., 2006).

***Silent cerebral infarcts (SCI):*** people with SCA who have SCI are at an increased risk of overt stroke by a 14-fold increase (Pegelow et al., 2001; DeBaun et al., 2012b). One challenge is that most children in low- and middle-income countries (LMICs) will not have MRI screening for SCI.

***Blood pressure:*** high blood pressure is a risk factor for stroke in children with SCA. Hypertension can cause injury to small cerebral blood vessels, making them more prone to rupture and leading to hemorrhagic stroke (Ohene-Frempong et al., 1998; Fox et al., 2022).

### Stroke prevention in children with SCA

Stroke in children with SCA is a significant health burden worldwide. Stroke prevention is essential in reducing the incidence and morbidity of stroke. The STOP (Stroke Prevention in Sickle Cell Anemia) trial demonstrated that regular blood transfusions for children with SCA and abnormal Transcranial doppler (TCD) velocity resulted in a 92% relative risk reduction in strokes when compared to no treatment (Lee et al., 2006). Barriers to blood transfusion in general are logistics reasons which includes taking time off work by caregivers, children missing school, transportation challenges to mention view. Others are negative experience on obtaining and maintaining venous access, iron overload and adherence to chelation agents, alloimmunization, fears and trepidation (Schlenz et al., 2022). However in low and middle income countries (LMICs), in addition to earlier are transfusion reactions, cultural beliefs, cost, and safety concerns, thus the need for alternative strategies for primary stroke prevention other than blood transfusion. A feasibility trial to determine the acceptability and safety of oral hydroxyurea (HU) therapy for primary stroke prevention in children with abnormal TCD measurements was conducted in Kano, Nigeria [SPIN (Stroke Prevention in Nigeria): NCT01801423] (Galadanci et al., 2017). The trial demonstrated high participant recruitment, retention, and adherence rates for HU. A new regional standard of care for stroke prevention was established using HU at 20 mg/kg/day (Galadanci et al., 2017). The SPIN trial also showed that the death rate was lower in children SCA receiving HU than in those not receiving HU, confirming HU safety in this malaria endemic region. Additionally, a phase 3 trial (SPRING trial, NCT02560935) compared low (10 mg/kg/day) vs. moderate (20 mg/kg/day) dose HU for stroke prevention (Pegelow et al., 2001); the trial showed no difference in stroke incidence among children treated with low dose HU compared with moderate dose HU (Abdullahi et al., 2023), though, those on moderate dose HU had a lower incidence of hospitalization for pain (DeBaun et al., 2012b).

The American Society of Hematology 2020 guidelines on cerebrovascular complications in SCD recommended that children with SCD and abnormal TCD measurements living in LMICs, where regular blood transfusions are not readily available, receive at least moderate dose HU (20 mg/kg/day) for primary stroke prevention (DeBaun et al., 2020). This recommendation was prior to the publication of the SPRING trial in 2022 that provided support for the use of low dose HU (10 mg/kg/day) for stroke prevention.

### Transcranial doppler ultrasound

TCD is a diagnostic tool that uses ultrasound waves to measure blood flow to and within the brain. TCD is recommended as a screening method for all children between 2 and 16 years of age with SCA, allowing for the identification of children at high risk of strokes, such as those with elevated blood flow velocities in the internal carotid artery (ICA) and middle cerebral artery (MCA). In the STOP trial the risk is classified as Normal risk (NR): Time Averaged Mean Maximal Velocity (TAMMV) <170 cm/s. Conditional risk is between 170 to <200 cm/s and abnormal is ≥200 cm/s. Children with SCD and abnormal TCD have a 17-fold increase in the risk of ischemic stroke when compared with those that have normal values (Adams et al., 1997). This strategy offers the opportunity for primary stroke prevention (Adams et al., 1992). Availability of TCD for children with SCD in Nigeria and other LMICs has been limited though we and others (Lagunju et al., 2015, 2021; Itanyi et al., 2020) have been part of an effort to train personnel and expand the use of TCD screening in children with SCD.

### Current practices for stroke prevention in SCA in Nigeria

Advances in primary and secondary stroke prevention for children with SCA in Nigeria have involved a combination of medical interventions, raising awareness of stroke, education and training of healthcare providers, and research (Galadanci et al., 2014; Ghafuri et al., 2022). These efforts are expected to reduce the incidence of stroke and improve the overall quality of life of individuals with SCA. Prior to the primary stroke prevention trials [SPIN (Galadanci et al., 2017) and SPRING (Abdullahi et al., 2022)] and a secondary prevention trial (SPRINT) that compared 10 and 20 mg/kg/day of HU to prevent recurrent strokes (Abdullahi et al., 2023), in Nigeria, experience and access to TCD for children with SCA in most of our facilities was rudimentary or non-existent. These clinical trials in Northwestern Nigeria made use of TCD (Bello-Manga et al., 2022a) as a screening tool for stroke prevention in children with SCA across Kano and Kaduna states. This was made possible due to international and local efforts in conducting effective research, training of staff, and provision of TCD machines across the two states (Ghafuri et al., 2022).

Evidence-based practice for primary stroke prevention in children with SCA involve screening for abnormal TCD velocities and if present, regular blood transfusion for at least 1 year (Lee et al., 2006). Oral HU therapy was studied as an alternative to regular blood transfusion in Africa due to challenges of blood transfusion in LMICs, and HU was found to be equally effective at low dose (10 mg/kg/day) compared to moderate dose (20 mg/kg/day) for both primary and secondary stroke prevention (Galadanci et al., 2017; Abdullahi et al., 2023). Apart from conducting stroke prevention research in children with SCA, it is important to translate evidence-based studies into clinical practice (Bello-Manga et al., 2022b). Integrating research findings into usual care of patients was enhanced by conducting TCD screening (Bello-Manga et al., 2024), demonstrating HU acceptability for both families and healthcare providers, and achieving regional government-aided support in providing free HU to children with SCA for primary and secondary stroke prevention; these factors were all downstream effects of the prior trials (Bello-Manga et al., 2022b). Although TCD screening is now standard of care in two Nigerian states, there is still a low reach for TCD screening (Galadanci et al., 2019; Bello-Manga et al., 2022a) with very few radiologists available to perform TCDs. Physicians rather than technicians perform and read ultrasounds in this region; an alternative strategy is needed to enable more children with SCA to receive TCD screening. Hence, we are studying the feasibility of a task-shifted stroke prevention program in a community hospital (clinical trial NCT05434000) where nurses are trained to complete TCD ultrasounds (Bello-Manga et al., 2022a). In addition to the use of TCD, comprehensive disease management is crucial for stroke prevention in children with SCA, starting with newborn screening to identify high-risk children, which is currently evolving in Kaduna state (Inusa et al., 2018). Regular follow-up visits in SCD clinic allow screening for profound anemia and prompt treatment when needed, including treatment of iron deficiency anemia. Ensuring adequate hydration, managing pain crises and addressing acute anemia and infection may prevent stroke in children with SCD. Meningitis secondary to *Streptococcus pneumonia* can cause stroke in the general pediatric population (Pryde et al., 2013) but whether or not this is an important cause of stroke in SCD is controversial (Kehinde et al., 2008).

Educating healthcare professionals about the risk factors for stroke in this population of children is critical. Nurses education is required to allow task shifting of TCD screening (Bello-Manga et al., 2022a) and incorporating SCD education into the routine curriculum of nurses and community health workers (CHEWS) is vital. Educating children and their caregivers about SCA, stroke risk factors, and the importance of treatment adherence is ongoing. Empowering families with knowledge and providing ongoing support can help them make informed decisions and maintain optimal care for children with SCA.

### Importance of early stroke detection in children with SCA

Global prevalence of stroke in SCA varies and without primary prevention is about 11% in France and USA by 19 years of age (Connes et al., 2013). In Africa, overall stroke prevalence in children with SCA is reported as 4.8% with a higher prevalence of 8.4% using WHO criteria which is a clinical diagnosis of stroke or transient ischemic attack (TIA) (Lagunju and Brown, 2012). Lagunju et al. (2019) in Ibadan, Nigeria recorded a hospital-based stroke prevalence of 6.8% in children with SCA. A stroke registry in the states of Kano and Kaduna, Nigeria was set up after the stroke prevention trials to follow up participants and determine the incidence of stroke (Abdullahi et al., 2022); thus additional data on stroke prevalence in Nigeria may be forthcoming. The lower prevalence in Kaduna state is presumably due to poor stroke detection. Reasons for this low detection of strokes may include lack of awareness among health care workers, families of children with SCA, and other members of the community as well as superstitious and cultural beliefs in some rural areas of Nigeria that recognize SCD as reincarnation, thus medical care may be avoided for children with SCD (Nzewi, 2001). Finally, universal newborn screening for SCA in most Nigeria States still rudimentary, combined with the high mortality rate among children with SCD <5 years of age, possibly >50% (Grosse et al., 2011), suggests some young children with SCA and stroke die without a diagnosis.

### Clinical presentation of acute stroke

Children with SCA are at high risk of developing stroke, and early diagnosis and treatment may limit the injury to the brain. However, acute stroke detection is a major challenge. Education on the common signs and symptoms of stroke in SCA as listed in Table 1 (Adams et al., 1998; Kirkham and DeBaun, 2004; Switzer et al., 2006) is required.

**Table 1:** Symptoms and signs of acute stroke in children.

• Sudden weakness or numbness on one side of the body or face, often face and arm together
• Difficulty speaking or understanding speech
• Loss of vision in one eye, vision to one side (visual field), or double vision
• Sudden severe headache
• Dizziness, loss of coordination, or difficulty walking
• Seizures, typically involving one side of the body
• Loss of consciousness (typically with hemorrhagic stroke)

Children with stroke may have several of these symptoms at the same time. Strokes can be ischemic or hemorrhagic, and the symptoms may not always be typical. Generally, stroke evaluation and diagnosis should involve the following steps.

#### Clinical evaluation

Obtain a thorough medical history, including any previous episodes of stroke, neurological symptoms, or known risk factors related to SCA. A physical examination with careful, age-appropriate neurological examination is essential to assess for signs of stroke, such as weakness, numbness, speech difficulties, and changes in consciousness. Using the Pediatric NIH Stroke Scale (PedNIHSS) is a recognized and standardized method of assessing a patient's neurological status (Ichord et al., 2011). The PedNIHSS is a validated pediatric acute stroke scale, a quantitative measure of stroke-related acute neurologic deficit which has proven intra- and inter-rater reliability (IRR) (Ichord et al., 2011); the NIHSS for children and adults takes 5–10 min to perform.

### Diagnosis of stroke

#### Neuro-imaging studies

In LMICs, neuroimaging is often not available, and WHO clinical criteria for stroke is utilized for stroke diagnosis. If available, MRI is the preferred imaging technique for confirming stroke in children because it provides detailed images of the brain (Jordan and Hillis, 2011) and can reveal both acute and chronic stroke lesions. Magnetic resonance angiography (MRA) can be used to assess blood vessels in the brain and detect abnormalities or narrowing (stenosis) that may increase stroke risk. In some cases, computed tomography (CT), or computed Tomography Angiography (CTA) scans may be used to assess for brain hemorrhage, ischemic stroke and can assess collaterals (Dundamadappa et al., 2020).

#### Laboratory investigations

A complete blood count will evaluate the patient's hemoglobin level, white blood cell count, and platelet count. Confirm the presence of hemoglobin S (HbS) in patients with sickle cell anemia using Hb electrophoresis and the percentage of HbS via HPLC if feasible (Kassim et al., 2015). Other investigations include markers of systemic inflammation, serum electrolytes, and clotting studies such as international normalized ratio and partial thromboplastin time if a hemorrhagic stroke is suspected. Tests such as electroencephalography, malaria parasites, and lumber puncture may be needed to exclude other mimics of acute stroke including seizure with post-ictal paralysis and serious infection.

### Challenges in diagnosis and treatment of stroke in Nigeria

Diagnosing stroke in children with SCA is challenging; stroke is often under-recognized by healthcare providers (Jordan and Hillis, 2011). In a Canadian study, ischemic stroke was only suspected in about 38% of children at the initial assessment (Rafay et al., 2009). This is partly due to non-specific symptoms like headache, seizure, and hemiparesis which may represent stroke or may be due to other conditions. Non-availability and high costs of neuroimaging investigations including CT and MRI are challenging in LMICs. Stroke identification in children from LMIC depends largely on careful clinical evaluation including a history and physical examination to identify any signs and symptoms of stroke. Neurological evaluation using the PedNIHSS (Ichord et al., 2011) will rapidly assess the severity of an ischemic stroke. Other challenges include poor knowledge of stroke in children and difficulties in stroke detection if the neurological findings are subtle.

### Challenges in treatment of acute stroke in SCA

Treatment of acute stroke in children with SCA typically involves a pediatrician, pediatric hematologist, neurologist, and physical therapist. The first goal is to minimize ongoing injury to the brain and the second is to prevent stroke recurrence. There is an evidence-based guideline for the treatment of acute stroke, TIA and silent infarcts in children with SCD (DeBaun et al., 2020). The adapted checklist in use in our center is outlined in Table 2.

**Table 2:** Checklist for acute stroke care in children with SCD in Northern Nigeria.

**Step**	**Action**
i.	Immediate assessment by the doctors on duty with the use of PedNIHSS to confirm the clinical diagnosis of stroke (Ichord et al., 2011).
ii.	Notify the hematology team and obtain vascular access.
iii.	The ASH guideline panel *recommends* prompt blood transfusion within 2 h of acute neurological symptoms.^*^
iv.	Exchange blood transfusion or modified exchange transfusion with group compatible HbAA blood within 2 h of presentation.^*^
v.	If Hb is <8.5 g/dl or exchange blood transfusion is not feasible within 2 h of presentation, then, an initial simple transfusion should be given and followed with an exchange or manual exchange blood transfusion.^*^
vi.	Maintain oxygen saturation >95% (pulse oximetry)
vii.	If febrile, the patient may require antimalaria, antipyretics, or antibiotics after investigating appropriately.
viii.	Monitor and maintain normal blood pressure.
ix.	If dehydrated give intravenous fluids 5% dextrose with 0.9% Saline + allow oral intake once swallowing function has been assessed as normal
x.	Surgical consultation in cases of hemorrhagic stroke.
xi.	Assess vital signs every 2 h for the first 24 hours.
xii.	Commence physiotherapy and hydroxyurea before discharge.

^*^A simple transfusion is usually possible for children with SCD and acute stroke. Ongoing transfusions and exchange transfusions are often not possible in Northern Nigeria.

Delay in recognition of stroke emphasizes the need for parental education focused on stroke awareness and early presentation for medical care. Delays in diagnosis occur even among healthcare providers, often due to stroke mimics such as migraine and focal seizure being more common than stroke (Rafay et al., 2009; Srinivasan et al., 2009). There are also often delays in treatment after presentation to the hospital and stroke recognition e.g., availability of blood transfusion and out of pocket expenses (Gabis et al., 2002). The high cost of pediatric stroke care is another important challenge. In North America the mean cost of acute stroke treatment was estimated as $15,003 in 2003 and the cost was found to be higher in children with hemorrhagic stroke (Perkins et al., 2009). In Nigeria, the majority of parents/caregivers have difficulty paying for care of minor ailments, and there is no country-wide health insurance. Thus, the costs of stroke-related care are greater than most families can afford, given that more than 40% of the Nigerian population live on <$381.75 per year (World Bank, 2020).

## Recommendations

In Nigeria and other LMICs, we must continue to increase awareness for stroke in SCD among healthcare providers and in the community to facilitate early detection. Expanding the reach of TCD screening by training nurses or other members of the healthcare team to allow increased primary stroke prevention is a critical short-term goal. Our team is working with state governments to develop policies that will support effective implementation of screening programs for stroke in children with SCD. Our long-term goal is to improve access to specialized care for children with SCD in both rural and urban communities which will aid in stroke prevention, early diagnosis, and prompt care. Further we hope that utilizing telemedicine technology to facilitate remote monitoring for children with SCD in areas with limited access to specialize care will be possible in the future.

## Author contributions

KA: Writing – review & editing, Writing – original draft, Conceptualization. HB-M: Writing – review & editing. LJ: Writing – review & editing.
